# Effects of Overt and Relational Bullying on Adolescents’ Subjective Well-Being: The Mediating Mechanisms of Social Capital and Psychological Capital

**DOI:** 10.3390/ijerph191911956

**Published:** 2022-09-21

**Authors:** Wenyan Hu, Yuhang Cheng, Ruoyu Du

**Affiliations:** 1 School of Marxism, Zhejiang Gongshang University, Hangzhou 310018, China; 2 Department of Sociology, Zhejiang University, Hangzhou 310058, China; 3 Department of Education, Practice and Society, University College London, London WC1E 6BT, UK

**Keywords:** school bullying, subjective well-being, social capital, psychological capital, adolescents

## Abstract

Based on the social and psychological capital framework, this study aimed to investigate the direct effect of bullying on adolescents’ subjective well-being and to reveal the potential psychosocial mechanisms in this relationship. Through the multi-stage cluster random sampling procedure, a cross-sectional survey was conducted among 728 adolescents from Hebei Province in China. Structural equation modeling was adopted for data analysis. After controlling for sociodemographic variables, the results indicated that only relational bullying had a significant negative effect on adolescents’ subjective well-being. Moreover, social capital and psychological capital mediated the relationship between relational bullying and adolescents’ subjective well-being. This study expands our understanding of the influencing mechanisms from bullying victimization to subjective well-being and also provides practical implications for future social policy development and relevant interventions.

## 1. Introduction

School bullying occurs widely around the world and has become a significant social concern [1,2]. According to the global report released by UNESCO in 2019, about 32% of adolescents have experienced school bullying [3]. In China, school bullying is also severe. As a survey conducted among primary and secondary schools in ten provinces and cities of China indicates, about 32.5% of students have experienced school bullying [4]. Previous research has shown that school bullying negatively affects the healthy development of adolescents in multiple dimensions, such as physical, psychological, and academic aspects [1,5,6]. Specifically, bullying victimization may cause physical injury to students, aggravate their loneliness and depression, foster delinquent behaviors such as alcohol abuse and smoking, and even jeopardize their learning motivation and academic performance [1,2,7]. Given the severe threats that school bullying may impose to student development, many scholars have begun to focus on the impact of school violence on adolescent well-being in recent years, especially in Western countries such as the United States, the UK, and Sweden [8,9,10].

However, in the social context of China, there remain several research gaps. Firstly, empirical studies that focus on exploring the potential influencing paths of school bullying by using quantitative methods are relatively lacking. Secondly, previous studies have shown that the impact of school bullying has significant differences among different age groups, and it normally achieves its peak during adolescence [11]. The high-school period, in which teenagers are aged 15–18, is a crucially important maturational and developmental process. *The Research Report on School Violence Cases* issued by the Supreme People’s Court of China illustrates that adolescents in high school are most likely to experience school bullying compared with children in other stages of their life [12]. However, past studies have rarely paid attention to them. Furthermore, school victimization encompasses both explicit modalities, such as physical aggression, and implicit forms, such as relational rejection and isolation. The impact on adolescent well-being may vary according to different types of school bullying, but only a few studies considered this topic in-depth [13,14]. Moreover, there is still a large gap between the theoretical paradigm and empirical evidence on the influencing pathways of school bullying on adolescent well-being, making it difficult for practitioners to find a breakthrough when dealing with cases related to school bullying. Considering this social reality and the current research context, this study aims to address the following two main questions: (1) Does the effect of school bullying on subjective well-being vary by the type of school bullying among high school-aged adolescents? (2) In addition, what are the influencing pathways underlying this relationship?

### 1.1. School Bullying and Subjective Well-Being

In this study, school bullying is defined as aggressive behavior that occurs on and off campus between students, in which the perpetrator intentionally bullies the victim in various ways. School bullying can manifest in various ways and is mainly divided into overt and relational bullying, depending on the nature of the bullying [5]. Overt bullying is a relatively intuitive and visual form of bullying, such as physical aggression (e.g., hitting and kicking) and verbal bullying (e.g., swearing and insults). By contrast, relational bullying is an indirect and implicit form of bullying in which the perpetrator harms the victim by manipulating their social relationships, mainly through social isolation and exclusion [15,16].

According to previous studies, both types of bullying styles can cause harm to the growth of adolescents. However, previous studies have found inconsistent effects [5], with some studies suggesting that the effects of overt bullying on the victim’s mental health were greater than those of relational bullying [17], while others demonstrated that the effects of relational bullying were more significant than those of overt bullying [1,15]. Notably, the studies mentioned above have only focused on victims’ adverse psychological outcomes (e.g., depression) instead of paying attention to their general well-being. In recent years, the importance of “subjective well-being” has come to the forefront of scholarship. Subjective well-being can be understood as an individual’s subjective judgment of their current state of life and evaluation of their life quality [18,19,20,21,22,23]. It has become an essential indicator of adolescents’ mental health status. Moreover, numerous previous studies have demonstrated that school bullying exacerbates adolescents’ psychological and behavioral problems and decreases adolescents’ life satisfaction as well as subjective well-being [1,24]. In the face of these research controversies and considering current research trends, this study further explores the differences in the effects of overt and relational bullying on adolescents’ subjective well-being. Accordingly, our hypotheses are as follows.

**Hypothesis** **1a.** *Overt bullying is negatively related to adolescents* *’ subjective well-being,*

**Hypothesis** **1b.** *Relational bullying is negatively related to adolescents* *’ subjective well-being.*

### 1.2. The Role of Social Capital and Psychological Capital as Mediators

Guided and inspired by prior research that attempted to explain the complex mechanisms between school bullying and adolescent well-being, another purpose of this study is to further explore and validate potential pathways that may help to expand the current understanding in academia [25,26,27]. The social and psychological capital theoretical framework [28] provides an integrated analytical perspective to uncover potential pathways. Based on the view of the theory, school bullying not only directly affects the victim’s subjective well-being but also impairs the individual’s social capital (the resources and support that individuals receive based on their social network) and psychological capital (the positive psychological qualities associated with the individual’s intrinsic characteristics), which indirectly endangers the victim’s subjective well-being.

According to Bourdieu’s (1986) definition, social capital is deemed as the resources and support that adolescents can obtain from their social network system [29]. From the view of social capital, school bullying, as a traumatic event in adolescents’ school life, reduces the level of trust in social relationships and decreases their perception of resources and support available in their relational networks, which in turn endangers students’ subjective well-being [15,30,31]. Additionally, according to the definition of psychological capital by Luthans et al. [32], psychological capital can be understood as the positive psychological qualities associated with individual traits. Based on the psychological capital perspective, previous studies have also demonstrated that bullying reduces children’s and adolescents’ resilience, sense of self-worth, and self-esteem, which in turn leads to more psychological problems and jeopardizes adolescents’ subjective well-being [1,27,33].

It is noteworthy that the majority of the current research focuses on only one dimension of social or psychological capital but lacks an integrated analysis and validation of the two types of capital. Moreover, the conceptual framework of social and psychological capital was proposed mainly in the context of workplace bullying in Western culture. Although some studies have validated the theoretical applicability in the context of school bullying [34], it still needs to be examined in more cross-cultural empirical studies. Thus, our hypotheses are as follows.

**Hypothesis** **2a.** *Social capital mediates the relationship between overt bullying and subjective well-being.*

**Hypothesis** **2b.** *Social capital mediates the relationship between relational bullying and subjective well-being.*

**Hypothesis** **3a.** *Psychological capital mediates the relationship between overt bullying and subjective well-being.*

**Hypothesis** **3b.** *Psychological capital mediates the relationship between relational bullying and subjective well-being.*

### 1.3. Present Study

**Figure 1:**
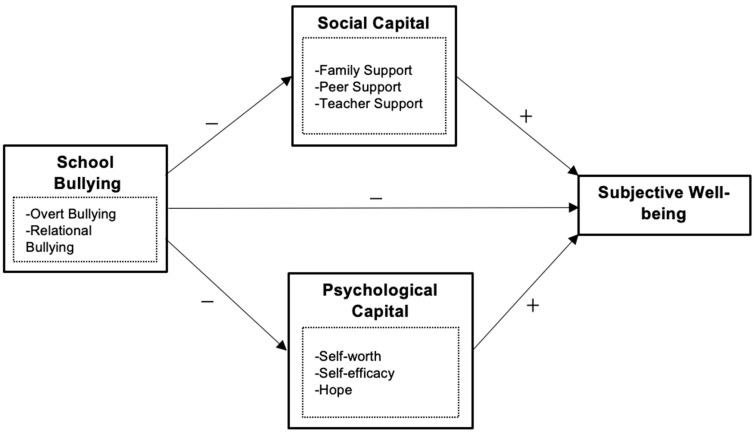
Theoretical framework.

Based on the above analysis, the literature review illustrates that even though the interrelationship between school bullying and subjective well-being has been widely tested in different cultural settings, research addressing the controversies between the impacts of different types of bullying is lacking. Furthermore, the potential mediating role of social capital and psychological capital has yet to be validated in an integrated framework focused on a group of high-school-aged adolescents. This study aims to address the research gaps and provide solid empirical evidence for future studies and practices. Accordingly, this study proposes the following theoretical framework (see Figure 1).

## 2. Materials and Methods

### 2.1. Sample and Procedure

Based on multi-stage cluster random sampling, which requires researchers to divide the population into groups (clusters) at various stages for better data collection, firstly, a list of schools in one city of Hebei Province in China was obtained by the research team, and four high schools were randomly selected. Secondly, one class was randomly selected from each grade (Grades 10–12) in each selected school. Then, all students in each selected class were invited to participate in our survey. Overall, we finally recruited 728 school-aged adolescents from four high schools as the research sample for this cross-sectional study. The study strictly followed various research ethics, such as anonymity, confidentiality, and voluntary participation. Research ethics approval of this study was received from the Research Ethics Committee of The Zhejiang Gongshang University, where the first author is affiliated. Before starting the survey, the researcher gave a detailed introduction to the student participants, their parents, and the school authorities about the purpose and content of the study and informed them of their right to participate voluntarily or to withdraw at any time. In the sample of this study, there were 345 males (47.4%) and 383 females (52.6%). Their average age was 16.33 years old (SD = 1.00). The proportions of junior (n = 157), sophomore (n = 359), and senior (n = 152) high-school students were 21.6%, 49.3%, and 20.9%, respectively. Most participants (719) were of Han ethnicity (98.8%). Slightly more than half of all participants (482) were from rural households (66.2%), and the remaining 246 teenagers were from urban households (33.8%). As for their family economic status, 9 teenagers were from extreme-poverty families (1.2%), 50 teenagers were from poor families (6.9%), 471 participants were from general families (64.7%), 177 teenagers were from wealthy families (24.3%), and 21 participants were from very well-off families (2.9%).

### 2.2. Measurements

#### 2.2.1. Bullying Victimization

The present study was conducted by using the Overt Victimization and Relational Victimization subscales of the Problem Behavior Frequency Scale developed by Farrell et al. [35]. The Overt Victimization and Relational Victimization subscales of the Problem Behavior Frequency Scale were developed to measure school bullying among youth. Each of the Overt Victimization subscales (e.g., being hit by other students) and the Relational Victimization subscale (e.g., having a classmate intentionally exclude you from an activity) contained five items. Scale options range from 1 (never) to 5 (always). Higher scores indicate higher levels of bullying. In this study, the items in the two subscales were used as secondary observed variables to measure the latent variables of overt bullying and relational bullying, respectively. The scale showed high reliability in this study, with internal consistency coefficients α of 0.866 and 0.923 for the overt bullying and relational bullying scales, respectively.

#### 2.2.2. Social Capital and Psychological Capital

The mediating variables in this study were social capital and psychological capital. The Multidimensional Scale of Perceived Social Support developed by Zimet et al. [36] was used to measure the available resources embedded in adolescents’ social relationships. On the basis of ecosystem theory, family, peers, and teachers are the main sources of social support for adolescents. The scale has three dimensions and 12 items measuring family support (e.g., I can get emotional support and help from my family), peer support (e.g., my friends can really help me), and significant other support (e.g., someone will be there for me when I have problems). The four items under the “significant others support” subscale were adapted to make them more applicable to the adolescent by using teacher support (e.g., my teacher is there for me when I have problems). The scale is based on a 5-point Likert scale, unless otherwise indicated, and scale options range from 1 (strongly disagree) to 5 (strongly agree). In this study, the mean scores of the three subscales were calculated and used as the observed variable to construct the latent variable of social capital. The family support (Cronbach’s alpha = 0.794), peer support (Cronbach’s alpha = 0.854), and teacher support (Cronbach’s alpha = 0.852) scales showed high reliability in this study.

In the present study, psychological capital was operationalized as the sense of self-worth, self-efficacy, and hope. Self-worth was measured through the Self-Concept Scale developed by Shevlin et al. [37]. The scale contains four entries, namely, “I feel like a failure”, “I feel worthless”, “I often feel ashamed for no reason”, and “I feel guilty for the things I have done or not done”. In this study, a 5-point Likert scale was used to assign values to the above scales. For statistical analysis, the four items were reverse-assigned, with higher scores indicating a higher level of self-esteem among adolescents. The internal consistency coefficient of the scale was 0.797, which shows high reliability. Self-efficacy was measured by using the General Self-Efficacy Scale (GSES) [38]. The scale contains 10 items (e.g., I can always solve problems if I try) and is scored on a 5-point Likert scale: from “totally disagree” to “totally agree”. The higher the score, the higher the adolescents’ self-efficacy level. The internal consistency coefficient of the scale in this study was 0.781. The measure of hope was based on the Hope Scale developed by Snyder et al. [39]. The scale contains six items (e.g., past life experiences will be helpful to me in the future) and was measured on a 5-point Likert scale, with higher mean scores indicating higher levels of hope among adolescents. In this study, the scale showed high internal consistency reliability (Cronbach’s alpha = 0.816). In this study, the latent variable of psychological capital was constructed using the mean scores of the self-worth, self-efficacy, and hope scales as secondary observed variables.

#### 2.2.3. Subjective Well-Being

The dependent variable in this study was subjective well-being, which was measured by the WHO Well-Being Index (WHO-5) [40]. The scale contains five items: “I feel cheerful in good spirits”, “I feel calm and relaxed”, “I feel active and vigorous”, “I woke up feeling fresh and rested”, and “My daily life has been filled with things that interest me”. The scale is scored on a 5-point Likert scale from “1 = strongly disagree” to “5 = strongly agree”. The mean scores of the five scale items were used to measure the adolescents’ subjective well-being, with higher scores indicating higher levels of subjective well-being. The scale showed high internal consistency reliability (Cronbach’s alpha = 0.829) in the sample of this study.

#### 2.2.4. Confounding Variables

The confounding variables in this study included gender (0 = female; 1 = male), age, household registration (0 = urban; 1 = rural), ethnicity (0 = minority; 1 = Han), and family economic status (1 = very poor; 5 = very rich).

### 2.3. Data Analytical Strategies

Firstly, this study conducted a descriptive statistical analysis and correlation analysis among key variables in SPSS 24.0 (IBM, Armonk, NY, USA). To verify the three research hypotheses mentioned above, this study used structural equation modeling in Amos 25.0 (IBM, Armonk, NY, USA) to validate the mechanisms of school bullying on adolescents’ subjective well-being. The validation of the structural equation model was conducted in two specific steps: the measurement model and the structural model. The measurement model was tested based on confirmatory factor analysis (CFA) to explore whether each latent variable (overt bullying, relational bullying, social capital, and psychological capital) in this study could be constructed by its corresponding indicators. Further, to test the direct effect of school bullying on subjective well-being and the mediating effect of social capital and psychological capital, this study tested hypotheses through two structural models. To test Hypotheses 1a and 1b, a “direct effect model” was developed to verify the direct effects of the two types of bullying on adolescents’ subjective well-being after controlling for the covariates. To test Hypotheses 2a and 2b and Hypotheses 3a and 3b, a “mediated effect model” was developed to verify the significant effects of the two mediating variables: social capital and psychological capital. The Sobel test and bootstrap analysis were used to determine the significance of the mediating effects. The chi-square (χ^2^), comparative fit index (CFI), and root mean square error of approximation (RMSEA) were used to verify the fit of the measurement model and the structural model. Due to the sensitivity of the chi-square values to the sample size, a good model fit is achieved if CFI > 0.90 and RMSEA < 0.08 [41,42].

## 3. Results

### 3.1. Descriptive Statistics and Bivariate Correlations

As shown in Table 1, the mean scores for relational bullying were higher than those for overt bullying. Peer support had the highest mean score among the three types of social capital. All variables were significantly correlated with each other, except for the correlation between overt bullying and teacher support (*p* > 0.05). Specifically, overt bullying and relational bullying showed significant negative correlations with all indicators of social and psychological capital; both types of bullying were also significantly and negatively correlated with adolescents’ subjective well-being. In addition, social capital and psychological capital showed significant positive correlations with subjective well-being.

### 3.2. Test of the Measurement Model

The measurement model results showed a positive model fit: χ^2^ = 443.687, df = 98, *p* < 0.001, CFI = 0.940, and RMSEA = 0.070. In addition, all observed variables loaded significantly on their corresponding latent variables. The factor loadings of the scale items on the latent variables of overt bullying and relational bullying ranged from 0.700 to 0.815 and 0.789 to 0.879, respectively; family support, peer support, and teacher support, as the three indicators of the latent variable of social capital, had factor loadings of 0.708, 0.569, and 0.505, respectively. Self-worth, self-efficacy, and hope, as the three indicators of the latent variable of psychological capital, had factor loadings of 0.708, 0.569, and 0.505, respectively. The factor loadings of all observed variables were higher than 0.50, which met the criteria for acceptable factor loadings of the measurement model, indicating that the measurement instruments used in this study could accurately measure the concepts and variables related to them.

### 3.3. Test of the Structural Model

#### 3.3.1. Direct-Effect Model

The direct-effect model was designed to validate distinct bullying typologies’ direct effects on adolescents’ subjective well-being. The direct-effect model reported a good model fit: χ^2^ = 397.941, df = 82, *p* < 0.001, CFI = 0.939, and RMSEA = 0.073. As shown in Table 2, after controlling for relevant demographic variables, relational bullying had a significant negative effect on adolescents’ subjective well-being (β = −0.213, *p* < 0.001); however, overt bullying did not have a significant effect on adolescents’ subjective well-being (β = −0.049, *p* > 0.05). In the direct model, only two confounding variables, gender (β = 0.085, *p* < 0.05) and family economic status (β = 0.134, *p* < 0.001), were significantly associated with adolescents’ subjective well-being. Thus, Hypothesis 1b was supported according to the empirical results, while Hypothesis 1a was rejected.

#### 3.3.2. Mediating-Effect Model

Based on the analysis of the direct model described above, the mediating-effect model further incorporated two mediating variables: social capital and psychological capital. The model fit indicators of the mediated-effect model also showed a good model fit: χ^2^ = 621.645, df = 194, *p* < 0.001, CFI = 0.933, and RMSEA = 0.055. The analysis results and model parameters of the mediated-effect model are detailed in Table 3.

We adopted the Sobel test and bootstrap analysis to verify whether the mediating effects of social capital and psychological capital were significant. The results of the Sobel test for the path “relational bullying → social capital → subjective well-being” showed that social capital played a significant mediating role in the relationship between relational bullying and subjective well-being (Z = −2.07 < −1.96, *p* < 0.05). The Sobel test for the path “relational bullying → psychological capital → subjective well-being” also indicated that relational bullying indirectly affected adolescents’ subjective well-being through the mediating mechanism of psychological capital (Z = −3.23 < −1.96, *p* < 0.01). After including mediating variables, the direct effect of relational bullying on subjective well-being was not significant (β = −0.016, *p* > 0.05). Thus, social and psychological capital played a fully mediating role. Specifically, relational bullying reduced adolescents’ social capital (β = −0.214, *p* < 0.01) and psychological capital (β = −0.281, *p* < 0.001), and decreases in social capital (β = 0.183, *p* < 0.01) and psychological capital (β = 0.563, *p* < 0.001) further negatively affected adolescents’ subjective well-being. In addition, the results of the bootstrap analysis also demonstrated the total indirect effects of relational bullying on subjective well-being via social capital and psychological capital (b = −0.230, 95% CI [−0.390, −0.103]), and these effects were significant. Based on these results, both Hypotheses 2b and 3b were supported.

## 4. Discussion

### 4.1. Summary of Empirical Results

First of all, this study found that only relational bullying had direct and indirect effects on adolescents’ subjective well-being among high-school-aged adolescents, suggesting that relational bullying is more harmful to adolescents than overt bullying. This finding is generally consistent with previous empirical studies conducted in the social contexts of East Asia, suggesting that there was no significant association between overt bullying and children’s mental health [15,43]. Considering the prevalent collectivist culture in East Asia, which places a high value on harmonious interpersonal relationships [44], overt bullying is clearly at odds with the collectivist value of harmony and is more likely to be noticed and intervened in by relevant people (e.g., parents and teachers). In contrast, the more subtle form of relational bullying is less likely to be detected by others. Consequently, relational bullying occurs more commonly and lasts longer, which could be one reason to explain why relational bullying is more harmful than overt bullying [15]. In addition, previous research has shown that the type of bullying continues to change with the age of the individual. Overt bullying is more prevalent among children in their early years and childhood. However, with the development of cognitive and emotional abilities, children tend to use more sophisticated social strategies than physical and verbal aggression when bullying others in order to avoid the punishment associated with direct bullying [45]. Especially during the high-school period, when the competition for group status among peers increases, peer relationships and interactions are extremely important for adolescents [46]. Adolescents may resort to relational bullying to maintain their status and affiliation in peer groups. Thus, as children enter adolescence, overt bullying declines and relational bullying rises [5,47,48], and the shift in the form of bullying explains why relational bullying has a more significant impact on the subjective well-being of high-school adolescents than overt bullying.

In addition, this study explains the mediating effect of external environmental resources (social capital) and intraindividual resources (psychological capital). This study demonstrated that although relational bullying had a direct negative effect on adolescent well-being, the direct effect of relational bullying on subjective well-being was not significant when the mediating variables of social capital and psychological capital were included, indicating that social capital and psychological capital fully mediated the relationship between relational bullying and subjective well-being. In the cultural context of collectivism, intergroup interdependence becomes an important way to acquire social and psychological capital. When adolescents are isolated from their peer groups, their perceived social resources will decrease along with their psychological capital, which is mainly reflected in the form of lower levels of self-efficacy, self-worth, and hope. The reduction in external social support and the strengthening of internal psychological vulnerability further impair adolescents’ evaluation of their own state of well-being and reduce their subjective well-being.

### 4.2. Theoretical Contributions

First, grounded in the analytical framework of social and psychological capital theories, the current study reveals the differences in the effects of overt and relational bullying on adolescents’ subjective well-being. Second, our study verifies the mediating effects of social and psychological capital in the relationship between school bullying and adolescents’ subjective well-being. In general, the findings enrich the theoretical explanation of the influencing pathways and provide empirical evidence for the future development of social policy and practical interventions.

### 4.3. Practical Implications

The above findings provide important empirical evidence and practical insights for future school bullying prevention. In recent years, China has issued a series of policies and regulations to deal with the problem of school bullying. For example, *The Notice on Special Treatment of School Bullying* issued by the Education Supervision Commission of the State Council of China in 2016 provides clear guidance on the prevention and treatment of school bullying, requiring schools around the country to strictly manage and punish this problem. This indicates that school bullying has become an important issue of concern at the national policy level. However, due to the hidden nature of relational bullying, the “perpetrators” of relational bullying are not easily screened by relevant disciplines, and it is also hard for educators to notice the “recipients” of relational bullying. The emotional and psychological damage and abuse caused by this kind of hidden violence may be more traumatic to students’ growth and development. Therefore, in the “blind spot” of policy coverage, school social workers and educational practitioners should work together to intervene with young victims of relational bullying to reduce the negative impact of hidden bullying on their well-being.

Furthermore, concerning the mediating roles of social and psychological capital, social-work-practice interventions need to take both the social environment and the individual’s own developmental characteristics into account. On the one hand, school social workers could use resource linkage to help isolated and excluded adolescents to obtain more external support from the multidimensional social system. On the other hand, school social workers could also help empower students through casework or group work, enhance their recognition of their own value and ability, improve their psychological capital, and realize “helping others to help themselves”. Therefore, enhancing social and psychological capital is a key breakthrough to cut off the negative effects of school bullying, and it could be one of the important practical strategies to enhance adolescents’ subjective well-being and psychological health.

### 4.4. Limitations and Future Directions

There are still some limitations in this study, which need to be further considered for future studies. Firstly, the cross-sectional research design adopted in this study only allows for a preliminary exploration of the relationships between variables and does not allow for causal inferences. Future studies are encouraged to conduct a longitudinal research design to further validate the theoretical model proposed in this study. Moreover, the sample in this study is only from Hebei Province in China, which might not be nationally representative. Future studies could enhance the generalizability of the findings through a larger sampling size. In addition, although the current study examined the distinct nature of overt bullying and relational bullying with systematic analyses of their influencing pathways on adolescents’ subjective well-being, we only measured bullying between peer groups and ignored bullying and violence between teachers and students. Future research could further explore how teacher bullying affects the development of adolescents. Finally, when experiencing negative interpersonal problems (e.g., relational bullying), adolescents in collectivist cultures may be more cognitively and emotionally vulnerable than those from Western individualist cultures [14,49]. Therefore, the effects of different forms of bullying on adolescents’ subjective well-being may differ between the cultural contexts of the East and West. Future research may attempt to conduct cross-national studies to further explore the differences in bullying types among diverse cultural contexts.

## 5. Conclusions

This study tested the differences in the effects of overt and relational bullying on adolescents’ subjective well-being and confirmed that only relational bullying had a significant direct effect on adolescents’ subjective well-being among high-school-aged adolescents. Moreover, guided by the framework of social and psychological capital theories, this study further explored the mediating mechanisms of external environmental resources (social capital) and intraindividual resources (psychological capital). In line with our expectations, we found that social capital and psychological capital fully mediated the relationship between relational bullying and subjective well-being. Taken together, this study deepens the current understanding of the relationship between school bullying and subjective well-being among adolescents in academia. More essentially, based on the strong empirical evidence, this study further encourages continuous efforts for the future prevention of school bullying and provides practical suggestions on this issue.

## Figures and Tables

**Table 1:** Descriptive statistics and correlation analysis of core variables.

	Mean	SD	1	2	3	4	5	6	7	8	9
1. Overt Bullying	1.578	0.675	1								
2. Relational Bullying	1.607	0.790	0.641 **	1							
3. Family Support	3.791	0.901	−0.111 **	−0.188 **	1						
4. Peer Support	3.931	0.886	−0.141 **	−0.193 **	0.406 **	1					
5. Teacher Support	2.970	1.073	−0.054	−0.092 *	0.368 **	0.262 **	1				
6. Self-worth	3.586	0.822	−0.244 **	−0.292 **	0.233 **	0.183 **	0.195 **	1			
7. Self-efficacy	3.458	0.608	−0.161 **	−0.191 **	0.211 **	0.182 **	0.141 **	0.279 **	1		
8. Hope	3.517	0.607	−0.134 **	−0.183 **	0.247 **	0.236 **	0.226 **	0.366 **	0.453 **	1	
9. Subjective Well-being	3.336	0.817	−0.185 **	−0.237 **	0.353 **	0.233 **	0.296 **	0.406 **	0.301 **	0.491 *	1

Note: * *p* < 0.05, ** *p* < 0.01.

**Table 2:** Results of direct-effect model parameters.

			B	S.E.	P
Subjective Well-being	<---	Gender	0.139	0.062	0.025
Subjective Well-being	<---	Age	−0.023	0.03	0.448
Subjective Well-being	<---	Household registration	0.106	0.063	0.092
Subjective Well-being	<---	Ethnicity	0.152	0.263	0.563
Subjective Well-being	<---	family economic status	0.166	0.045	***
Subjective Well-being	<---	Overt bullying	−0.065	0.09	0.467
Subjective Well-being	<---	Relational bullying	−0.235	0.071	***

Note: B: unstandardized path coefficient; S.E.: standard error; P: significance level; *** *p* < 0.001.

**Table 3:** Results of mediating-effect model parameters.

	Social Capital			Psychological Capital			Subjective Well-Being		
	B	S.E.	P	B	S.E.	P	B	S.E.	P
Gender	−0.006	0.062	0.920	0.091	0.043	0.032	0.049	0.055	0.376
Age	−0.039	0.030	0.198	0.012	0.020	0.554	0.026	0.026	0.332
Household Registration	−0.025	0.064	0.694	−0.027	0.043	0.524	0.139	0.056	0.012
Ethnicity	0.395	0.265	0.136	0.091	0.180	0.611	−0.029	0.232	0.902
Family Economic Status	0.209	0.046	***	0.120	0.032	***	0.000	0.041	0.992
Overt Bullying	0.001	0.090	0.995	−0.069	0.061	0.261	0.004	0.079	0.960
Relational Bullying	−0.192	0.072	0.007	−0.175	0.050	***	−0.018	0.064	0.783
Social Capital							0.226	0.069	0.001
Psychological Capital							0.997	0.120	***
R^2^		0.107			0.165			0.469	

Note: B: unstandardized path coefficient; S.E.: standard error; P: significance level; *** *p* < 0.001.

## Data Availability

Not applicable.
